# Voluntary Saccade Training Protocol in Persons With Parkinson’s Disease and Healthy Adults

**DOI:** 10.3389/fnagi.2019.00077

**Published:** 2019-04-05

**Authors:** Paul B. Camacho, Ronald Carbonari, Sa Shen, Cindy Zadikoff, Arthur F. Kramer, Citlali López-Ortiz

**Affiliations:** ^1^Department of Kinesiology and Community Health, University of Illinois at Urbana–Champaign, Urbana, IL, United States; ^2^Beckman Institute for Advanced Science and Technology, University of Illinois at Urbana–Champaign, Champaign, IL, United States; ^3^Center on Health, Aging and Disability, University of Illinois at Urbana–Champaign, Champaign, IL, United States; ^4^Department of Neurology, Northwestern University Feinberg School of Medicine, Chicago, IL, United States; ^5^Center for Cognitive and Brain Health, Department of Psychology, Northeastern University, Boston, MA, United States; ^6^Joffrey Ballet Academy, The Official School of the Joffrey Ballet, Chicago, IL, United States

**Keywords:** eye movements, saccades, Parkinson’s disease, training, voluntary saccades, healthy adults, saccade latency, saccade amplitude

## Abstract

**Background:** Voluntary saccade function gradually decreases during both the progression of Parkinson’s disease (PD) and neurologically healthy adult aging. Voluntary saccades display decreased length and increased saccade latency, duration, and the number of compensatory saccades in aging and PD. Saccades serve as the key eye movement for maintaining salient features of the visual environment on the high visual acuity fovea of the retina. Abnormal saccade behavior has been associated with freezing of gait in PD. We have not identified any studies that have investigated improvement in voluntary saccade function using voluntary saccade training.

**Objective:** We report an experimental protocol that tests a training paradigm following the principle of specificity to improve voluntary saccade velocity and amplitude, while decreasing latency and the number of compensatory saccades.

**Methods:** Persons with PD (n = 22) and persons with no known neurological disorders (n = 22) between the ages of 40 and 65 years will be recruited. In a randomized-block study design, all participants will perform voluntary saccades to targets in eight cardinal and intercardinal directions. In each of the eight sessions during the four-week intervention period, participants will train at three target amplitudes. Participants will perform 40 trials for each amplitude block, consisting of five randomly presented repetitions for each direction. Voluntary and reflexive saccades will be recorded pre- and post-intervention, along with clinical mobility assessment using the Movement Disorder Society Unified Parkinson’s Disease Rating Scale. Mobility scores, the amplitude, latency, and duration of the first saccade, and the number of saccades to reach the fixation target will be analyzed using an ANOVA of mixed effects.

**Discussion:** This protocol holds promise as a potential method to improve voluntary saccade function in persons with PD. Should persons with PD not improve on any outcome following the intervention, this lack of response may support the use of saccade assessment as a response biomarker for the diagnosis of PD.

**Trial Registration:** This protocol was retrospectively registered at ISRCTN (ISRCTN.com) since July 25, 2018. The first participant was recruited March 12, 2016. The protocol identifier is 17784042.

**Descriptive Title:** A two-arm, pre/post-protocol to compare the effects of a four-week voluntary saccade training intervention in persons with Parkinson’s disease and healthy adults aged forty years or older.

## Introduction

### Voluntary Saccade Function Impairment in Parkinson’s and Healthy Aging Adults

Visual function is a major area of decline in Parkinson’s disease (PD) progression and healthy aging adults (Terao et al., 2011; Dowiasch et al., 2015). One element of visual function that has been correlated with PD progression and neurologically healthy aging by a number of studies is saccade behavior (Terao et al., 2011; Dowiasch et al., 2015; Seferlis et al., 2015; Noiret et al., 2017). Saccades are the ballistic eye movements that allow the high acuity fovea of the retina to quickly orient toward salient elements of the visual environment. Persons with PD exhibit lower saccade amplitude, higher saccadic latencies, and longer durations to achieve targets (Mosimann et al., 2005; Terao et al., 2011). Deficits in voluntary saccade control contribute to mobility impairments related to turning during navigation (Ambati et al., 2016; Nemanich and Earhart, 2016). It has been noted that turning during navigation in persons with PD increases instability and risk of falling (Stack and Ashburn, 1999). Voluntary saccade function deficits are also present to a lesser extent in neurologically healthy aging adults (Chen and Machado, 2016; Fernandez-Ruiz et al., 2017).

### Existing Treatments

Various treatments, including electrical brain stimulation, have been suggested for improving voluntary saccade function in PD (Chen and Machado, 2016). An eye-movement training protocol consisting of horizontal saccades to increasingly distant points and cognitively more challenging targets, such as letters and words, was shown to increase reading speed in older adults with age-related macular degeneration (Seiple et al., 2005). Other treatments include the anti-saccade paradigm, which requires the suppression of a saccade to a stimulus and voluntary saccade to a location of equal eccentricity in the opposite direction (Jamadar et al., 2015). However, such studies focus on accuracy and error rate and do not address hypometria as is needed for navigation (Jamadar et al., 2015; Ambati et al., 2016).

### Need for a Trial

Recent work has investigated the difference between smooth-pursuit performance in persons with PD and persons with no known neurological disorders (Ito et al., 2013; Fukushima et al., 2015). However, the current literature does not include voluntary saccade practice as a method of comparing potential improvements in voluntary saccade function for persons with PD and healthy aging adults. While we cannot predict if there will be immediate short- or long-term benefits to the participants in the study, exercising eye movements can potentially benefit eye movement control. As with any other movement training, exercise increases circulation to the muscles and improves their physiological function. Physical mobility is of the utmost importance for the quality of life in the general population. Furthermore, recent PD literature indicates that abnormal saccade behavior is associated with the freezing of gait and difficulty in navigation (Lohnes and Earhart, 2011; Ambati et al., 2016; Nemanich and Earhart, 2016; Stuart et al., 2017). Improving abnormal saccade behavior may affect freezing of gait and other motor-related PD symptoms. Improving these outcomes for persons with PD has the potential to improve the same daily activities as those in healthy aging persons, as well as increasing walking ability (Lohnes and Earhart, 2011; Stuart et al., 2017). Further, saccade function has been demonstrated as a key factor in the slowing of reading, decrease in driving abilities, and eye-hand coordination in several studies (Schmitt et al., 2015; Coats et al., 2016).

#### Saccade Biomarker Potential

Identifying non-invasive biomarkers of PD onset is imperative for disease treatment. Saccadic abnormalities have been suggested as a diagnostic tool for PD, including early stage progression marking and differential diagnosis from other tremor disorders such as essential tremor (Yerram et al., 2013). Saccade abnormalities may also be useful as markers for frontal cortex and basal ganglia malfunction and degeneration in PD, as well as changes in the cerebellum (Terao et al., 2013). van Stockum et al. (2012) later suggested that due to the differences in reports of saccadic latency deficiencies and facilitation, the hypometria seen across different saccadic paradigms may be a better measure for the effect of PD on saccades. Control of voluntary saccades has also been highlighted as a promising area of motor control in general (Baird-Gunning and Lueck, 2018). Diagnosis in the early stages will benefit individuals with PD, enabling prompt disease treatment and management to slow disease progression. In addition to the person diagnosed with PD, this also benefits their immediate family and support network.

One criticism against saccade measurements as a diagnostic tool for PD is that the abnormalities seen in saccadic movements tend to differ based on the experimental context (MacAskill and Anderson, 2016). Abnormalities in saccade latency and amplitude are known to occur in multiple neurodegenerative disorders, which could lessen biomarker specificity (MacAskill and Anderson, 2016). However, recent reviews outlined differential characteristics of saccadic performance in PD and several other neurodegenerative diseases (Termsarasab et al., 2015; MacAskill and Anderson, 2016). Additionally, research into the characteristic relationship between saccade duration, peak velocity, and amplitude has not been extensively undertaken in PD. This “main sequence” is believed to characteristically optimize the costs of speed and saccadic accuracy at a given amplitude (Bahill et al., 1975; Harris and Wolpert, 2006). If the main sequence relationship is affected by training differences in persons with PD compared to neurologically healthy persons, the biomarker potential for voluntary saccade training response would be further supported. One benefit of saccadic measurements as a PD biomarker is that assessments of eye tracking systems are non-invasive, unlike biochemical markers, which require blood or CSF extraction. Additionally, saccade testing could occur in a clinical setting without the need for physician supervision and biochemical testing facilities, with the most common side effect being possible fatigue from eye movement.

#### Risks/Benefits Comparison

Risks associated with eye training include the possibility of eye strain and orbital myositis, which is a rare autoimmune disorder that may result from inflammation due to vigorous exercise. These risks are minimal and reduced by the progressive periodization of amplitudes in the training regimen. The potential benefits of improved eye coordination, range of motion, and responsivity to visual stimuli outweigh the minimal risk for eye strain/injury. The greatest amount of risk in the study is associated with performing OFF-state assessments in participants with PD. The OFF-state occurs when the motor effects of levodopa and other dopaminergic medications are not present due to delayed intake of these medications. During the OFF-state, intensified motor symptoms that may be uncomfortable – such as dyskinesia, rigidity, spasticity, bradykinesia, dystonia, and freezing – may be present. However, discomfort and possible risk of falls are mitigated by limiting the inclusion criteria to only include individuals in the early stage of the disease (H&Y score 1–2; see Table 1 for criteria) where these symptoms are not severe in general or in the OFF-state. Neurologist clearance – as well as facilitation of dialogue between participant and neurologist, participant and researchers, and researchers and neurologists – will provide an informed environment for how to manage the two-hour disruption in medication. Further, risks of discomfort and falling will be minimized through the use of wheel chairs, gait belts, and secure seating during the eye-tracking assessment.

**Table 1:** Criteria for Hoehn & Yahr stages one and two.

H&Y: Stage One	H&Y: Stage Two
1. Signs and symptoms on one side only	1. Symptoms are bilateral
2. Symptoms are mild	2. Minimal disability
3. Symptoms are inconvenient but not disabling	3. Posture and gait affected
4. Usually presents with tremor of one limb	
5. Friends have noticed changes in posture, locomotion, and facial expression	

It is common practice to perform assessments in the OFF-state in the field of PD clinical research because the methodology provides a controlled environment for assessing the disease without the influence of movement-modifying medications that mask the disease condition. This is especially important in biomarker research of PD patients. Assessment in the OFF-state condition increases relevance and generalizability of the study evidence to populations with undiagnosed and untreated PD. Generalizability of results may lead to greater instances of early detection and allow for informed diagnoses of individuals not yet receiving movement-modifying medications. The increased applicability of data collected in OFF-state assessments far outweighs the risks.

### Saccade Training Time Selection

Due to the current lack of literature studying voluntary saccade training through practice, the training dose of 30 min, twice per week, for four weeks was chosen based on training doses seen in other eye training protocols (Fischer and Hartnegg, 2000; Bibi and Edelman, 2009; Jamadar et al., 2015; Knox and Wolohan, 2015; Kleiser et al., 2017; Johannesson et al., 2018). The limit of 30 min of practice per day was chosen to reduce the likelihood of eye strain.

### Explanation for Choice of Comparators

There will be no comparator intervention used in this study. The effects of the voluntary saccade training intervention in persons with PD will be compared to those in persons with no known neurological disorders so that we can determine whether the group with PD is more responsive to the intervention. A greater response in the participants with PD would indicate a significant improvement in saccade performance. A lack of response to the intervention would indicate a resistance to improvement in saccade performance that may be characteristic of persons with PD.

### Objectives

#### Research Hypothesis

We hypothesize that before and after comparisons of voluntary and reflexive saccade performance will show a greater decrease in latency and the number of saccades needed to reach the target, along with a greater increase in saccade amplitude and velocity in Parkinson’s disease participants as compared to participants with no known neurological disorders.

#### Study Objectives

##### Primary objective

To determine whether voluntary saccade training decreases voluntary latency, reflexive latency, and number of saccades needed to reach a target amplitude and increases saccade amplitude in persons with PD compared to persons with no known neurological disorders.

##### Secondary objectives

To determine whether training voluntary saccades affects motor disability in persons with PD. We will also investigate whether training voluntary saccades affects the relationship of the main sequence. Should statistical analysis reveal that these improvements are absent in participants with PD, we propose that saccade performance would be a potential early PD biomarker.

### Trial Design

This trial is designed as a two-arm, pre-post, single center pilot trial with an equal number of participants with PD and participants with no known neurological disorders (control), which will be analyzed using an analysis of variance (ANOVA) with mixed effects.

## Methods: Participants, Interventions, And Outcomes

### Study Setting

All data collection and participant training will be conducted in the Neuroscience of Dance in Health and Disability (NDHD) Laboratory at the University of Illinois at Urbana–Champaign. Access to the laboratory space is limited to the research team and participants, with a partitioned area for saccade training and eye tracking data collection. This protocol has been approved by the local IRB and all NDHD laboratory staff have completed the ethics and best practices training required by the local IRB and the University of Illinois. Due to the small size of this study, Champaign County, Illinois will be sufficient for the recruitment of both study groups. The adult population of Champaign County is approximately 170000 persons and is largely rural. We will attempt to representatively recruit participants according to the ethnicity data provided by the US Census Bureau: 72.4% White, 13.4% Black or African American, 0.4% Native American, 10.9% Asian, 0.1% Native Hawaiian and other Pacific Islander, 2.8% two or more races, and 6.0% Hispanic or Latino^1^.

### Eligibility Criteria

A final decision on inclusion will be made in consultation with the principal investigator once all screening materials are complete (see Figure 2 for a full timeline). During the initial contact interview, the research assistants will read a script describing the study and assessment procedures; if interested, they will be provided via email with the physician release form to be completed by the participant and physician along with the consent form for the participant’s review. Once all questions from the participant regarding the study procedures have been answered by the research team, the consent form is completed, and the physician release form is received via U.S. mail, email, or by hand and reviewed by the principal investigator, a determination will be made whether to proceed with screening using the MoCA and Modified Hoehn & Yahr levels. Only if all inclusion criteria are met, the participant will continue with the study (see Table 2 for eligibility criteria). Research shows that the prevalence of PD in men is 1.5 times greater than in women. Therefore, we expect that ratio will be reflected in our recruitment.

**Table 2:** Eligibility criteria for protocol.

**Inclusion Criteria**
1. To be medically stable with diagnosis of PD by meeting the United Kingdom PD Society Brain Bank Criteria OR- no known neuromuscular disorders for the for the control group.
2. To have a Modified Hoehn & Yahr stage 1–2 (with unilateral involvement only, unilateral and axial involvement, and bilateral involvement without impairment of balance) in the conventionally defined OFF medication state.
3. To have medical clearance form from their physician for participation in the study.
4. To be in a stable regimen of PD medication 30 days prior to the initiation of the study and until the completion of the study.
5. To be willing and able to provide informed consent.
6. To be of age 40 and up.
7. Must have a caregiver/family member present for OFF-state assessment sessions.
**Exclusion Criteria**
1. Presence of dementia based on The Montreal Cognitive Assessment (MOCA) – score of less than 25.
2. Diagnosis of comorbid neurological disorder such as epilepsy.
3. History of neurological injury such as stroke.
4. History of brain surgery such as deep brain stimulation.
5. Concurrent severe medical illness which in the opinion of the research team will preclude participation in the study (such illnesses may include but not limited to severe or uncontrolled cardiovascular disease, hypertension, pulmonary disease, or diabetes).
6. Inability to attend and participate in at least seven of the training sessions.
7. Uncorrected vision, history of retinal disease (e.g., macular degeneration), presence of optic neuropathy due to glaucoma or ischemic optic neuropathy, pseudoexfoliation syndrome, ocular surgery, ocular trauma, visually significant cataract, orbital myositis, blindness or refractive errors outside –5 to +3 D.
8. Indication by the participant s neurologist in the medical release form that testing the participant in the OFF-medication state would put PD participants at significant risk for medical complications.

### Voluntary Saccade Training Intervention

#### Equipment

Training and testing will be performed using the SR EyeLink II eye tracking system (SR Research). The EyeLink II system for assessments and training has two cameras on a head mount located directly below eye level. The pupil is tracked by a device that captures infrared light reflected off the lens and cornea of the eyes. The lens, cornea, and other parts of the eye absorb a small amount of energy from infrared light, but the energy is less than 18% of the Maximum Permissible Exposure level as certified by the American Standards Institute (ANSI Z 136.1-1973). The EyeLink II system will be mounted to the participant’s head using the adjustable straps, such that the head camera bar will be parallel to the display screen, on which four infrared emitters will be placed in a rectangle. The participant will be then positioned, using a chin rest support, so that the participant’s resting focus point will be at the center fixation target display on the screen in the middle of the rectangle of emitters. The experimenter will position the eye tracker on the participant’s head so that the participant can see targets displayed at the amplitude of interest. The chin rest also serves to isolate eye-movements from head movements during eye tracking and maintain a set distance from the display screen. A Sharp Aquos 178.5 cm by 100 cm television will be used as the display for the participant (Sharp Corporation, Sakai-ku, Sakai, Japan). This screen mirrors the computer display visible to the experimenter during training and testing sessions. A second host computer runs the SR Research data capture and processing during sessions. The host computer also displays pupil position and the view of the left and right eye cameras in real time. After adjusting the eye cameras and pupil threshold to track the pupils moving in the eight cardinal and intercardinal directions, the cameras will be then calibrated using the SR Research program. The experimenter will then perform a validation of the camera calibration prior to beginning a training or testing block.

#### Training

Over the course of the four-week intervention period, participants will undergo eight training sessions of 30 min each. During each training session, three target amplitudes will be trained on the six target amplitudes: 10°, 20°, 30°, 40°, 45°, and 50°. Each amplitude will be trained in two different training sessions over the course of the intervention period. This follows an intercalated training schedule of progressive and regressing load design (see Table 3). For each amplitude, the participants perform saccades to each of the eight cardinal and intercardinal directions. Each direction will be indicated five times for a total of 40 trials per amplitude per session. Participants will place their head on a chin rest 40 cm away from the screen in order to isolate the movement of the eyes. Training will involve a visual display designed to initiate only voluntary saccades. Training will begin with a warm-up for the eyes in order to reduce the possibility of injury. Participants will foveate on a central fixation starting point and wait for an instructional arrow that points to one of eight visual targets arranged in eight cardinal points in the perimeter of a circle.

**Table 3:** Training schedule of amplitudes. On both sessions of each week, the same three amplitudes will be trained.

Week	Amplitudes Trained
1	10°, 20°, 30°
2	10°, 20°, 40°
3	20°, 30°, 45°
4	20°, 40°, 50°

**Figure 1:**
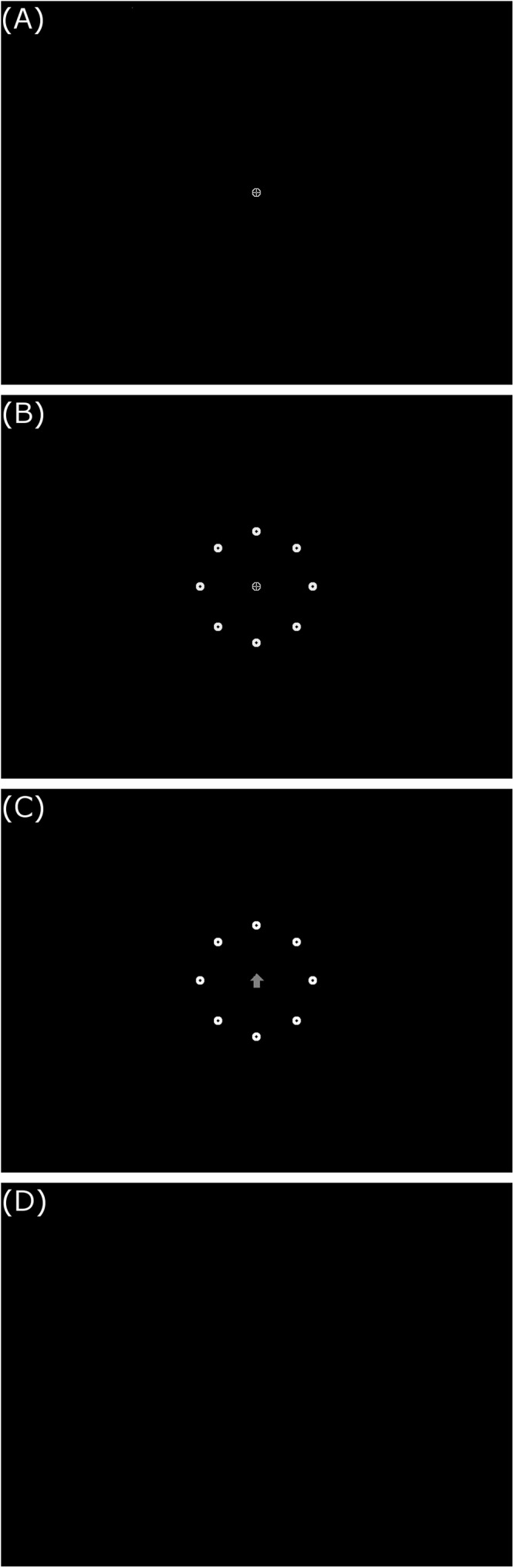
Example training/testing trial display sequence. **(A)** Central fixation target, **(B)** 20° target circle, **(C)** northern direction indication arrow, and (D) blank reset screen. All images presented in 800 × 600 resolution on screen.

Every trial consists of a fixation period, target circle presentation period, direction indication period, and resetting period (see Figure 1). During the fixation period the participant is instructed to focus on the center of a circular fixation bulls-eye target. Next, in the target circle presentation period, a circle of targets in each of the eight directions is presented around the central fixation target. The central fixation target is then replaced by an arrow indicating which direction to look in and fixate on the target there located. After the fixation is recorded at the indicated target, the resetting period follows with the presentation of the sole central fixation target. Due to the nature of mass-practice of eye movements, there is an inherent risk of eye fatigue or strain in participants. However, participants in this study will be instructed to rest at the first sign of any eye pain or fatigue, to minimize the risk and influence of strain or fatigue on the outcomes of the experiment.

**Figure 2:**
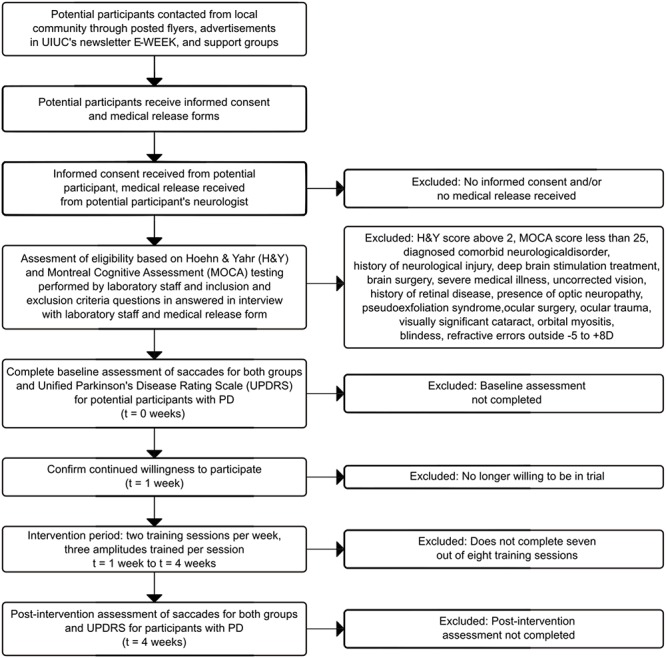
Participant flow timeline.

**Figure 3:**
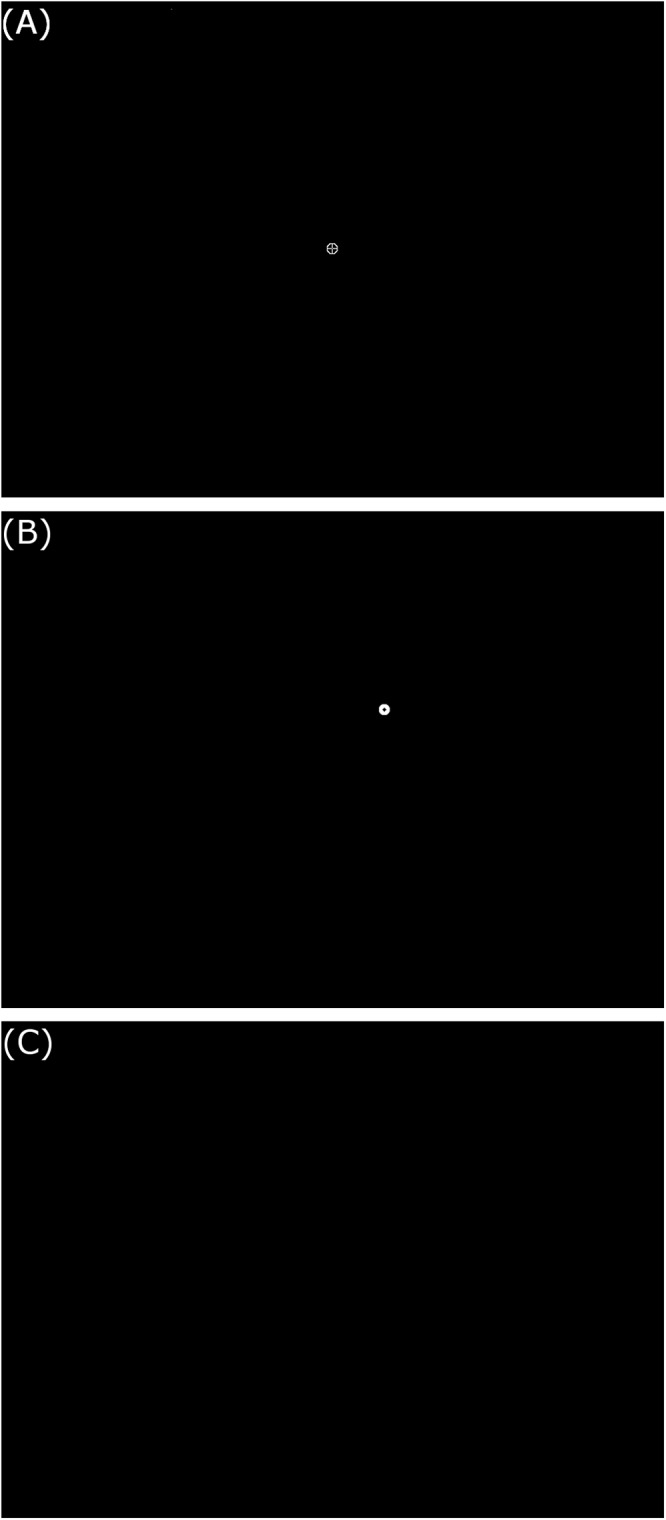
Example reflexive testing trial display sequence. **(A)** Central fixation target, **(B)** 20° northeast target, and **(C)** blank reset screen. All images presented in 800 **×** 600 resolution on screen.

Participants who are unable to complete seven of the eight training sessions due to fatigue or other events related to the intervention will be omitted from the study and receive prorated compensation. The intervention is designed to minimize fatigue by providing rest breaks regularly and as the participant indicates a need for rest. During both the training and testing sessions, eye tracking data will be collected by the SR Eyelink II system and stored. While only testing session data will be analyzed to determine the effects of the intervention, training session data can be used to check that training sessions will be completed per the protocol instructions. This check data includes the number of trials per direction per amplitude, amplitudes trained, and collection dates for each session.

### Modifications

In the unlikely case of an unexpected event, the research team, in consultation with the participant’s physician, will make the decision to modify or terminate the trial. As this is a pilot trial, there will be no adjustment to sample size in the course of the study.

### Concomitant Care

(1) Involvement in other interventional studies will not be permitted for participants in either group.
(2) Participants will continue their physician prescribed treatment during their participation.

### Outcomes

#### Primary Outcome Measures

The primary outcomes of this study are four measures of voluntary and reflexive saccade performance: (1) the normalized amplitude calculated as the amplitude of the saccade divided by the target eccentricity, (2) the mean normalized saccadic velocity calculated as the mean normalized amplitude divided by saccade duration, (3) saccadic latency calculated as the time from the presentation of the directional cue to the saccade onset. The saccade onset is calculated as the time between the presentation of the directional arrow and the first eye movement crossing the velocity threshold of 30°/s and the acceleration threshold of 8000°/s^2^ specified by the EyeLink^^®^^ II User Manual (Jainta et al., 2011). The fourth outcome is the number of saccades needed to reach the target. The first three outcomes are only calculated on the first saccade toward the target.

#### Secondary Outcome Measures

One secondary outcome for this study is Movement Disorder Society-sponsored new version of the Unified Parkinson’s Disease Rating Scale (MDS-UPDRS). The MDS-UPDRS assesses motor examination and complications, motor aspects of daily living, and non-motor aspects of daily living using a combination of questionnaire and task performance assessment (Goetz et al., 2008; Martinez-Martin et al., 2013). The MDS-UPDRS will only be assessed in participants with PD, as participants with no known neurological disorders should not present disability measurable with these instruments. Additionally, the peak saccadic velocity, duration, and amplitude will be analyzed as a measure of changes to the main sequence (Bahill et al., 1975).

Primary outcomes will be assessed by a member of the NDHD laboratory staff blinded to secondary outcome assessment, which will be performed on a separate day (see Table 5 for investigator team timeline). Secondary outcome assessors will be blinded to primary outcomes and will not be involved in training sessions. Changes after the four-week intervention from the initial value of each primary and secondary outcome measure will be analyzed using an ANOVA of mixed effects.

### Participant Timeline

Table 4 contains a full timeline of participant involvement.

**Table 4:** Timeline of participant involvement.

	Study Period					
	Enrolment	Allocation	Post-allocation			
Timepoint	*Week* –*1*	*Week 0*	*Week 1*	*Week 2*	*Week 3*	*Week 4*
**Enrolment:**						
Eligibility screen	X					
Informed consent	X					
Medical release	X					
Allocation		X				
**Intervention:**						
Parkinson’s group			X	X	X	X
Healthy group			X	X	X	X
**Assessments:**						
Clinical Screening		X				
Saccades			X			X
Clinical			X			X

#### Sample Size

There will be two groups with the same treatment: (1) a control group of participants with no known neurological disorders and (2) a test group comprised of participants with PD that have H&Y levels 1–2. In the absence of existing data in the literature to estimate the required same sample size for a protocol of this nature, the estimation is based on the number of eyes tested and trained in other eye movement studies and a 10% attrition rate seen in other studies performed by the authors (Hall et al., 2010; Knox and Wolohan, 2015; Ivanov et al., 2016; Heath et al., 2017).

### Recruitment

Persons with PD (*n* = 22) and neurologically healthy persons (*n* = 22), between the ages of 40 and 65 years will be recruited. Participants will be recruited from the local community through posted flyers, advertisements in UIUC s newsletter E-WEEK, and support groups (in a 60-mile radius around the UIUC campus).

## Methods: Data Collection, Management, And Analysis

### Saccade Function Testing

During the week prior to and the week following the intervention period, participants will be tested for both voluntary and reflexive saccade performance (see Table 6 for summary of saccade testing). Voluntary saccade testing will involve the same task as the training sessions, in the 10°, 20°, and 30° amplitudes only. In the reflexive saccade testing session, the same amplitudes will be tested following the reflexive testing protocol. As in the voluntary saccade testing, each amplitude will be tested for the eight cardinal and intercardinal directions. Five trials will be performed per direction, resulting in 40 trials per amplitude in a testing session. The structure of each trial consisted of the initial fixation period, followed by the simultaneous disappearance of the central fixation target and appearance of the reflexive target, and finally the resetting period. Unlike in the voluntary saccade trials, there is no presentation of the circle of targets prior to the appearance of the reflexive target and no arrow to indicate the direction of movement to be performed (see Figure 3). Assessments will begin with a warm-up for the eyes in order to minimize the risk of injury. Participants with no known neurological disorders (see Supplementary Material Data Sheet 2 sample physician release form) will complete only the eye-tracking related assessments. There will be one pre-training assessment and one post-training assessment, totaling two assessment periods for the control group protocol. Each assessment period should last no longer than 1.5 h including rest periods.

**Table 5:** Investigator team activity timeline.

			Approximate	Prestudy	Basline/			Intervention
	PD Only		time to	screening/	Intervention	Intervention	Intervention	week 4/
Activity/assessment	(Yes/	No)	Staff member	complete	(min)	consent	week 1	week 2	week 3	conclusion
Prescreening consent		Study coordinator	5	X				
Screening checklist		Study coordinator	10	X				
Consent form		Study coordinator	45	X				
Medical release and relevant medical history	Yes	Potential participant’s neurologist	30	X				
Hoehn and Yahr scale	Yes	Clinical assessors	30		X			
Montreal Cognitive Assessment	Yes	Clinical assessors	30		X			
Unified Parkinson’s Disease Rating Scale	Yes	Clinical assessors	30		X			
Voluntary saccade assessment		Staff member	45		X			
Reflexive saccade assessment		Staff member	45		X			
Voluntary saccade training intervention		Staff member	45		X X	X X	X X	X X
Termination form		Study coordinator	N/A					X
Serious adverse event form		Study coordinator	N/A	As needed throughout protocol		
Progress note		All team members	N/A	X	X	X	X	X
Communication log		All team members	N/A	Every phone or in-person contact outside of regular study visits		

**Table 6:** Saccade testing summary table.

VOLUNTARY	REFLEXIVE
**Amplitudes**: 10°, 20°, and 30°	**Amplitudes**: 10°, 20°, and 30°
**Directions (*Cardinal*):** North, Northwest, West,	**Directions (*Cardinal*):** North, Northwest, West,
Southwest, South, Southeast, East, Northeast [8 total]	Southwest, South, Southeast, East, Northeast [8 total]
**Repetitions PER Direction**: 5×	**Repetitions PER Direction**: 5×
**Total Trials**: 120	**Total Trials**: 120
**Task**: Voluntary Saccade	**Task**: Reflexive Saccade

*Combination of voluntary and reflexive eye-tracking related assessments will last approximately 1.5 h including rest time*.

Participants will perform eye-tracking related assessments in ON and OFF motor-related medication states in order to completely characterize the effects of eye-movement training in PD (see Table 7 for OFF-state timeline). Participants will continue regular intake of any medications that are unrelated to motor symptoms in PD.

**Table 7:** OFF-state testing timeline for participants.

Night Before	7:00 AM	8:30 AM	9:00 AM	10:00 AM
Take last dose of medication at 11 PM (taken 8 h before morning assessment)	Eye-Tracking Assessment (OFF-state)	MDS-UPDRS Assessment	**RETURN TO MEDICATION (60-min break)**	Eye-Tracking Assessment (ON-state)

### Clinical Mobility Testing for Participants With Parkinson’s

Following completion of eye-tracking related assessments, the participant’s motor function will be assessed using the Movement Disorder Society-United Parkinson’s Disease Rating Scale (MDS-UPDRS) in the OFF-state for an accurate description of PD stage. To achieve the OFF-state, participants will be instructed to take their last dose of medication approximately 8 h before their scheduled morning assessment. This approach will time the initiation of the OFF-state with the beginning of data collection. Immediately following the OFF-state eye-tracking assessment, the MDS-UPDRS will be administered to participants in the NDHD Laboratory which is equipped with ballet barres for support. Immediately after the MDS-UPDRS administration, participants will be instructed to take their medication and take a 60 min long break while they return to their normal ON-state. This protocol will mitigate the time spent in the OFF-state to approximately 2 h. Sixty-minutes after the intake of medication, participants will complete a final eye-tracking assessment in the ON-state. In total, pre/post-assessments will last at most 4 and a half hours, including the 60 min break and assessment beaks. A summary of an example Parkinson’s pre-training and post-training assessment timeline follows: If the participant takes medications in a schedule other than every 8 h, the participant will time the start of the experiment with their regular time of medication intake and withhold from taking their regular dosage for the first 2 h of the experiment. After those 2 h, the participant will resume regular medication intake.

### Processing

Eye tracking data for both the left and right eyes is first converted to ASCII files, which will be then converted into two types of ASCII files: gaze data, which consisted of pupil position relative to the room, and head referenced (*href*) data, which consists of pupil position relative to the head. In order to minimize false positive detection of saccades caused by head movement, the *href* data is then used for further processing. This data is then processed in MATLAB (MathWorks, Inc., Natick, MA, United States) using a program developed by Ronald Carbonari. This program converts eye tracking data from pixel measurements of movement distances to radial distance based on the fixed distance of the participant from the experiment display. The program also uses median pupil position during fixation to find the center of the visual focus for each trial to calculate real saccade distances, removing the assumption that the center of the visual fixation target is the center of actual fixation. Blinks and their subsequent movements will be detected and removed using a data-driven blink finder in the MATLAB program. Saccade latency is calculated as the time between presentation of the directional arrow and the first eye movement crossing the velocity threshold of 30°/s and the acceleration threshold of 8000°/s^2^, as specified by the EyeLink^^®^^ II User Manual (Jainta et al., 2011). The program then removed faulty fixations and appended the start of each saccade to occur 1.6 ms after the end of the previous fixation. This auto-cleaned data will be then stored and plotted, allowing the data to be visualized for each trial and cleaned on a per trial basis using an additional section of the MATLAB script edited by the user. This script allows users to, on either eye, delete trials missing excessive position data or delete false saccades not detected by the automated cleaning process. Additionally, users could shift the start and/or end of a fixation to match visually determined correct times based on the plot of radial position versus time. Saccades are automatically shifted along with the endpoints for adjusted fixations. The user can also move individual saccades and insert fixation-saccade pairs to match visually determined significant pupil movements. Only the first three saccades in each trial will be analyzed since this was a sufficient number of saccades to reach the target in preliminary tests.

### Data Management

All participants will be assigned a code for de-identification for all data collected. The participant code will be stored in a locked cabinet and destroyed when the study procedures are completed. Data analyses will be conducted on the coded non-identifiable data. All data will be kept in a locked file cabinet or in encrypted, password protected research computers. We will retain all screening data for those who qualify and volunteer and destroy the screening data for those who are excluded or do not choose to participate in the study. The informed consent, medical clearance, and verification of PD diagnosis will be stored together in a locked cabinet.

### Statistical Methods

After data cleaning is completed using the automatic and user-guided portions of the MATLAB program, the cleaned data for all trials, which contains pupil position data for both eyes, will undergo statistical analysis. Using SAS, an ANOVA with mixed effects will be performed using the finalized data concerning four variables of interest for both voluntary and reflexive saccades: (1) normalized mean velocity, (2) normalized angular distance, (3) latency for the first saccade in a trial, and (4) saccade count to target (SAS Institute Inc., Cary, NC, United States). For participants with PD, the UPDRS scores before and after the intervention will be included with the eight saccade variables in the ANOVA with mixed effects to determine if any significant clinical mobility improvements occur. In secondary analyses, saccade-related measurements will be compared between the voluntary and reflexive saccades and by target amplitude. In the case of non-normality, non-parametric statistical methods will be used. Saccade data that are not viable for statistical analysis due to problems in recording eye movements, such as blinks obscuring saccades and loss of fovea tracking during a saccade initiation or endpoint, will not be included in the statistical analyses. Main sequence analysis will be included as a secondary form of analysis. Due to the comparatively weaker relationship seen between peak velocity and amplitude in the range that will be recorded, analysis of the relationship between mean velocity and amplitude and the relationship between duration and amplitude will be included (Bahill et al., 1975).

## Methods: Monitoring

### Data Monitoring

Due to the low risk of this protocol, no data monitoring committee is required.

Should the intervention cause orbital myositis or other physical injuries in any of the participants, the research team, in consultation with the patient’s physician, will make the final decision to terminate the trial.

### Harms

Data collected with the eye tracker during assessments in the study will not give an immediate indication about participant risk. However, participant comfort and concern for their own health will be continuously monitored verbally by research personnel throughout the assessment. If the participant expresses concern all research procedures will stop and research personnel will call 911 with the participant’s consent. In case of such an adverse event the IRB will be immediately notified. The participants are required to have a caregiver/family member present during OFF-assessments. There will be an area on the physician clearance form (Supplementary Material Data Sheet 3 sample physician release form for PD) for instructions to minimize risk during the OFF-state as well as a schedule for the administration of medications before and after the OFF period. The medical waiver will include an additional area for any special instructions to research assistants as necessary. Patients that could be at significant risk for medical complications by withdrawing from medications will be excluded from the study.

### Auditing

The trial will be audited by compliance entities associated with the University of Illinois at Urbana–Champaign system.

## Discussion

Improvement in voluntary saccade function may occur due to changes at multiple levels of the saccade. Saccade improvements may occur due to increased eye muscle strength as well as better nervous system planning and control of saccades. Three sets of extraocular muscles are involved in performing saccades depending on their component composition (Sparks, 2002). The medial and lateral rectus muscles generate horizontal saccade movements (Sparks, 2002). The superior and inferior rectus muscles function with the superior and oblique muscle pairs for production of vertical saccade components (Sparks, 2002). Production of oblique saccades requires all three pairs of muscles to coordinate together (Sparks, 2002). Functional improvement of these muscles may impact the ability to perform movements of different horizontal and/or vertical components depending on which muscles are affected. At the nervous system level, communication between areas involved in the generation of saccades may increase to produce better coordination between the horizontal and vertical saccade systems. Control over peak saccadic velocity may also change as the training task aims to facilitate larger voluntary saccadic amplitudes, potentially inducing a change to the main sequence that has been seen previously in voluntary control of reflexive saccades (Muhammed et al., 2018).

The superior colliculus (SC) has been identified as a key area for target and timing selection for saccades in primates (Port and Wurtz, 2009). Neurons in the SC are thought to form a map of saccades by direction and amplitude, requiring the activity of a group of SC neurons to form a saccade of the correct amplitude and direction (Sparks, 2002). SC neurons receive cortical and subcortical input and communicate output to the midbrain and pontine areas, as well as all premotor areas associated with control of eye and head movements (Sparks, 2002). The cortical frontal eye fields – while not necessary for saccade initiation – contribute to saccade control, through activity mediated by the SC (Sparks, 2002). The cerebellum plays a key role by creating the error signal for saccades, which contributes to saccade accuracy, speed, and lack of variability (Robinson and Fuchs, 2001). This signal is also generated by different areas of the cerebellum depending on whether the saccade has a horizontal or vertical direction (Robinson and Fuchs, 2001). The posterior vermis and caudal fastigial nucleus generate horizontal saccade signals, while vertical saccade information is handled by the interpositus nucleus (Robinson and Fuchs, 2001). Through these signals, the cerebellum adds correction information to saccade commands prior to initiation of movement to achieve accurate saccades of the necessary speed (Robinson and Fuchs, 2001).

A lack of response to voluntary saccade training in the primary or secondary outcome measures would support the use of this protocol for testing saccades as part of a biomarker assessment for early detection and diagnosis of PD. An added dimension of biomarker potential may come from a differential response of the main sequence to the training between the two groups as measured by changes in characteristic peak velocity for given saccadic amplitudes. This would create a more robust saccade-based biomarker by adding a treatment-response dimension to the characterization suggested as a diagnostic tool by previous studies (Terao et al., 2013; Yerram et al., 2013; Termsarasab et al., 2015).

## Conclusion

This intervention holds significant promise as a low-cost, low resource-demand tool to improve motor functions of the eyes in persons with PD and adults with no known neurological disorders as well as mobility outcomes associated with eye function in persons with PD. Should persons with PD not show improvements on any metric following the intervention, a lack of response to voluntary saccade training may support saccade assessment as a biomarker for early detection and diagnosis of PD.

## Ethics and Dissemination

### Research Ethics Approval

All methods have been approved by the local IRB committee (see Table 8 for a full IRB revision history as of the publication of this article). All subjects will provide written informed consent (see Supplemental Materials Data Sheet 1 sample informed consent for informed consent form) in accordance with the Declaration of Helsinki. This protocol has been designed in accordance with SPIRIT guidelines for clinical trial protocols (see Table 9 for a summary of SPIRIT-required trial registration information).

**Table 8:** IRB protocol revision chronology.

**Original Submission**	July 2, 2015	
Approval date	January 15, 2016	
Amendment 01	February 16, 2016	Update of research team list.
Amendment 02	October 3, 2016	Change in research location to Freer Hall room 250.
Amendment 03	July 31, 2017	Addition of testing in the off-medication state for participants with PD.
Amendment 04	April 9, 2018	Update of research team list.
Amendment 05	July 19, 2018	Medical release form specific to healthy controls added.

**Table 9:** Trial registration data.

Primary registry and trial identifying number: 17784042
Date of registration in primary registry: July 25, 2018
Secondary identifying numbers: N/A
Source(s) of monetary or material support: Unfunded
Primary sponsor: University of Illinois at Urbana–Champaign
Secondary sponsor(s): None
Contact for public queries: CLO, Ph.D., MA lopezort@illinois.edu
Contact for scientific queries: CLO, Ph.D., MA University of Illinois at Urbana–Champaign
Public title: Voluntary saccade training in persons with Parkinson’s disease and healthy adults
Scientific title: Voluntary saccade training in persons with Parkinson’s disease and healthy adults – two-arm, pre/post-trial
Countries of recruitment: United States of America
Health condition(s) or problem(s) studied: Parkinson’s disease, voluntary saccades, reflexive saccades
Intervention: Voluntary saccade training
Key inclusion and exclusion criteria: Ages eligible for study: ≥40 years; Sexes eligible for study: both; Accepts healthy volunteers: yes
Inclusion criteria: For participants with PD: (1) medically stable with diagnosis of PD by meeting the United Kingdom PD Society Brain Bank Criteria, (2) to have a Modified Hoehn & Yahr stage 1–2 (with unilateral involvement only, unilateral and axial involvement, and bilateral involvement without impairment of balance) in the conventionally defined OFF medication state, (3) to have medical clearance form from their physician for participation in the study, (4) to be in a stable regimen of PD medication 30 days prior to the initiation of the study and until the completion of the study, (5) to be willing and able to provide informed consent, (6) to be of age 40 and up, and (7) must have a caregiver/family member present for OFF-state assessment sessions.
For neurologically healthy participants: (1) no known neuromuscular disorders, (2) to have medical clearance form from their physician for participation in the study, (3) to be willing and able to provide informed consent, and 4) to be of age 40 and up.
Exclusion criteria: (1) presence of dementia based on The Montreal Cognitive Assessment (MOCA) –score of less than 25, (2) diagnosis of comorbid neurological disorder such as epilepsy, (3) history of neurological injury such as stroke, (4) history of brain surgery such as deep brain stimulation, (5) concurrent severe medical illness which in the opinion of the research team will preclude participation in the study (such illnesses may include but not limited to severe or uncontrolled cardiovascular disease, hypertension, pulmonary disease, or diabetes), (6) inability to attend and participate in at least seven of the training sessions, (7) uncorrected vision, history of retinal disease (e.g., macular degeneration), presence of optic neuropathy due to glaucoma or ischemic optic neuropathy, pseudoexfoliation syndrome, ocular surgery, ocular trauma, visually significant cataract, orbital myositis, blindness or refractive errors outside –5 to +3 D, (8) indication by the participant’s neurologist in the medical release form that testing the participant in the OFF-medication state would put PD participants at significant risk for medical complications.
Study type: Interventional
Allocation: no randomization; Intervention model: two-arm pre-post; Masking: non-masked
Primary purpose: eye movement training
Study Phase: Phase 0
Date of first enrolment: March 12, 2016
Target sample size: 44
Recruitment status: Recruiting
Primary outcome(s): For both voluntary and reflexive saccades: number of saccades needed to reach target amplitude, for first saccade: latency, normalized mean velocity, normalized amplitude
Key secondary outcomes: Unified Parkinson’s Disease Rating Scale

#### Protocol Version

IRB# 16033

Issue date: July 19th, 2018

Protocol amendment number: 06

Authors: Paul B. Camacho *(PBC)*, Ronald Carbonari *(RC)*, Sa Shen *(SS)*, Cindy Zadikoff *(CZ)*, Arthur F. Kramer *(AK)*, Citlali López-Ortiz *(CLO)*

### Protocol Amendments

Important protocol modifications will be approved by the local IRB and communicated to co-investigators, trial participants, trial registries, the clinical trial publishing journal.

### Consent or Assent

The written informed consent will be completed by the participant before enrolling in the study and undertaking baseline data collection. The participants will have the opportunity to read the informed consent and ask any questions about the procedures to the PI before participation in the study. We will provide the participant with a copy of the signed informed consent document. We will give a period of at least 24 h for review of the consent form. This will assure that the participant has had ample time in reviewing and understanding the consent form prior to signing. The PI will be available to answer any questions regarding the consent form.

### Confidentiality

All biographical and medical information about potential participants will be stored in paper form in a locked cabinet. We will retain all screening data for those who qualify and volunteer. We will destroy the screening data for those who are excluded or do not choose to participate in the study. All participants will be assigned a random identification number for all data collection. The participant identification code will be stored in paper form and destroyed when the study procedures are completed. Clinical measures taken to as part of the recruitment process and study will be deidentified, using assigned participant numbers, and completed in paper form. Raw eye tracking data will be collected and stored by participant number on a computer, which will have no internet connection. Access to the NDHD laboratory is restricted to trained staff and, during training or testing, the participants and their caregivers. At no point will anyone who is not a member of the NDHD staff have access to any collected data. Data analyses will be conducted on the coded non-identifiable data. The data will be kept for 5 years after publication, as required by the American Psychological Association. Only deidentified data will be released after the trial as part of peer-reviewed scientific journal articles and storage in a data repository.

### Access to Data

The final trial de-identified dataset will be available to members of the NDHD laboratory staff and stored in an online data repository. To minimize bias, investigators performing data processing of eye tracking data will not have access to clinical measure outcomes or identifying information until the end of the study. Similarly, clinical assessors will not have access to eye tracking data or other information that could bias assessment.

### Ancillary and Post-trial Care

Study participants will continue with regular, prescribed medical care throughout the experiment. In the event of physical injury, their physician will be contacted.

### Dissemination Policy

The proposed forms of dissemination are presentations at scientific conferences and publications in scientific journals. Only deidentified data will be released after the trial as part of peer-reviewed scientific journal articles and storage in a data repository. To be eligible for authorship, all potential authors must meaningfully contribute to and approve the final manuscript. All authors are expected to contribute to the shaping of the protocol and performance of some aspect of the study. The full protocol and statistical code will be accessible via an online data repository, along with the participant-level dataset for the purposes of study reproducibility.

## Sponsor Contact Information

Trial Sponsor: University of Illinois at Urbana–Champaign

Sponsor’s Reference: N/A

Contact name: Dr. Citlali López-Ortiz

Address: 221 Freer Hall 906 S Goodwin Ave. Urbana, IL 61801

Telephone: (217) 300-1022

Email: lopezort@illinois.edu

### Sponsor Responsibilities

The sponsor will have no direct role in study design, data collection, management, analysis, interpretation of data, writing of the report, or decision to submit the report for publication.

## Author Contributions

CL-O conceived the study and design and involved in all aspects of the study. AK supervised the experiments design. CZ assisted with medical aspects of the protocol design. PBC helped refine the study design and wrote the manuscript. RC set up the eye tracker and computing systems and developed the data processing software. RC and PBC helped with implementation. SS contributed with statistical expertise. All authors contributed to refinement of the study protocol, approved the final manuscript, and agreed to be accountable for the content of the manuscript.

## Conflict of Interest Statement

The authors declare that the research was conducted in the absence of any commercial or financial relationships that could be construed as a potential conflict of interest.

## Footnotes

1 https://www.census.gov/quickfacts/fact/table/champaigncountyillinois,US/PST120217
