# Supplementary material for: Voluntary Saccade Training Protocol in Persons With Parkinson’s Disease and Healthy Adults
Source: Front Aging Neurosci. 2019 Apr 5;11:77. doi: 10.3389/fnagi.2019.00077 (PMC6459894; doi:10.3389/fnagi.2019.00077)
Supplement: Supplementary file 1 [file Data_Sheet_1.pdf]

Department of Kinesiology and Community Health
Louise Freer Hall
906 South Goodwin Avenue
Urbana, IL 61801-3895

INFORMED CONSENT FORM AND AUTHORIZATION FOR RESEARCH

Title: Are Voluntary Saccades Robust Bio-markers for PD?

Principal Investigator: Citlali López-Ortiz, PhD, MA

You are being asked to take part in a research study. This document has important information about the reason for the study, what you will do if you choose to be in this research study, and the way we would like to use information about you and your health.

A description of this clinical trial will be available in ISRCTN.com. This Web site will not include information that can identify you. At most, the Web site will include a summary of the results. You can search this Web site at any time.

What is the reason for this study?

Parkinson's disease (PD) can lead to difficulties with eye movements. Since exercise has been proven effective for improved mobility in PD, we are investigating a training regimen that specifically targets eye movements.

What will you do if you choose to be in this study?

The study will consist of 45-minutes training sessions, two times per week for four weeks of training. Training will consist of completing eye movement exercises to various targets on a screen at progressively larger amplitudes. During this time, we will be recording your eye movement characteristics. You will also be asked to complete one pre and one post assessment.

Assessments

For the Control Group:

You will be asked to perform eye movements to targets on a computer screen for two pre/post assessments. While you are doing the eye movements, we will record eye movement characteristics. Each eye tracking assessment will measure voluntary and reflexive saccades, is seated the entire time, and should last around one and a half hours.

Each assessment will consist of a visit lasting around 2 hours and will take place at [redacted]

For only the Parkinson's Group:

You will be asked to perform eye movements to targets on a computer screen for one pre-assessment and one post-assessment. You will be performing these assessments in both an "ON" and "OFF medication state". Assessment days will be comprised of the following:

University of Illinois at Urbana-Champaign
Institutional Review Board

Approved January 25, 2019
Expires January 24, 2020
IRB #16033

- Eye tracking assessment in the "OFF-state"
- Movement assessment using the MDS-UPDRS in the "OFF-state"
- Eye tracking assessment in the "ON-state"

Eye Tracking Assessment: You will be asked to perform eye movements to targets on a computer screen in an "ON" and "OFF medication state". While you are doing the eye movements, we will record eye movement characteristics. Each eye tracking assessment will measure voluntary and reflexive saccades, is performed while seated the entire time, and should last around one and a half hours.

MDS-UPDRS: The MDS-UPDRS assesses movement and non-movement related clinical measures of Parkinson's. The MDS-UPDRS will be administered after the first "OFF-state" eye tracking assessment. Wheel chairs, gait belts, ballet barres, and research personnel will be available during these assessments.

Achieving OFF-state: Performing assessments in the "OFF-state" will provide the best characterization of training effects from eye-movement training. If you take your movement symptoms related medication every 8 hours, you will be asked to take your last dose of medication the night before, 8 hours prior to the scheduled morning assessment so that the masking effects of the medication are reduced by the time of the assessment.

If you take your movement related medications in a schedule other than every 8-hours, you will time the start of the experiment with the regular time of medication intake and withhold taking your regular dosage for the first two hours of the experiment. After those two hours you will take your medication and resume your regular medication intake

You need to consult with your neurologist regarding the safety for withdrawing from medications.

Assessments will last approximately 4.5 hours.

An example timeline is provided below but the start time may differ according to your personal medication intake schedule.

**Night Before**	**7:00AM**	**8:30AM**	**9:00AM**	**10:00AM**
**Take last dose of medication at 11PM**<br>**(taken 8 hours before morning assessment)**	Eye-Tracking Assessment<br>(OFF-state)	MDS-UPDRS Assessment	**RETURN TO MEDICATION**<br>**(60 minutes break)**	Eye-Tracking Assessment<br>(ON-state)

We require you to have a caregiver/family member present for "OFF-state" assessments to aid in transport, etc.

University of Illinois at Urbana-Champaign
Institutional Review Board

Approved January 25, 2019
Expires January 24, 2020
IRB #16033

Training (BOTH GROUPS)

For the training sessions, you will produce progressively larger eye movements on a television screen while we track your eye movements. Each training session will last thirty minutes and take place at [redacted] You must come in for training two times per week for a total of four weeks and complete eight training sessions. If a session is missed, you will be given an opportunity to reschedule.

Example Study Timeline

	MON	TUES	WED	THURS	FRI
Week 0	Assessment	Training		Training	
Week 1		Training		Training	
Week 2		Training		Training	
Week 3		Training		Training	Assessment

What equipment will be used?

Eye tracking assessments will be taken using Eye Tracker equipment.

The Eye Tracker system for assessments has two small cameras on a light, non-invasive head mount that hold the cameras directly below the level of your eyes. The eye movements are monitored by a device that captures infrared light reflected off the lens and cornea of the eye. The lens, cornea, and other parts of the eye absorb a small amount of energy from infrared light, but the energy is less than 18% of the Maximum Permissible Exposure level as certified by the American Standards Institute (ANSI Z 136.1-1973). In the case of eye movement training sessions, an array of cameras will be placed close to the computer screen and capture your eye movements in a similar manner. A TV monitor or a computer screen will be used to display the targets for the eye movements.

What are some of the risks and discomforts that may happen to people who are in this study?

With any sort of training there is risk of injury. Because we are targeting the eyes in our exercises, eye strain and soreness are possible. Eye strain is easily treated with rest and will normally go away after a couple minutes. There is a low-risk possibility, however, of developing orbital myositis, an inflammatory response of the eye muscles. **In order to mitigate these risks we will remind you take breaks at any time you feel discomfort or even end a training session early if you so choose. There will be rest breaks given every 3 to 5 minutes throughout the training session.**

If you have Parkinson's disease:

There is risk associated with withholding medication for a total of 2-hours from the due time to achieve an "OFF-state". Withholding medications can lead to discomfort and increased motor dysfunction such as dyskinesia, dystonia, muscular spasticity, and/or freezing. Increased motor dysfunction can lead to adverse events such as falling or accidental injury. Since you are in the early stages of PD. These risks are not considered high. However, to minimize potential

University of Illinois at Urbana-Champaign
Institutional Review Board

Approved January 25, 2019
Expires January 24, 2020
IRB #16033

falls or movement related discomfort on assessment days, you will be received at your car with a wheel chair and transported to [redacted] You may come with your caregiver/family member for every session to assist, but it is required for assessment days. While obtaining medical clearance from your expert neurologist, you should speak with your doctor about your risk associated with going off your medication regimen for two hours. You should also ask for specific instructions on how to schedule medication intake to withdraw/return to your normal regimen. The neurologist approval form has dedicated space for these purposes. If you become uncomfortable at any time during the "OFF" assessment, you are free to withdraw from the study and take your medication.

What are some of the benefits from participating in this study?

While we cannot predict if there will be direct benefits from participating in this study, you will be contributing to the long-term goal of investigating methods of rehabilitation for those with PD. If you take part in this study you will aid researchers in finding new biomarkers for PD as well as help in creating a better understanding for the disease. We hope that the results of this study will lead to a better quality of life for those with PD.

Are there any financial costs to participating in this study?

There are no financial costs to you.

Travel Expenses:

You will be reimbursed [redacted]

Will I receive payment for this study?

Yes. You will be compensated [redacted] for each training session of approximately 30min and [redacted] for each assessment session. We will cover the cost of meter parking while you participate in the study. For participation that does not reach 100% of the training sessions, payment will be prorated. You will receive payment at the end of your participation in the study.

You will fill out a form for the Accounting Services at University of Illinois giving your name, address, and Social Security Number in order to issue a check for your study participation and to be mailed by US Postal Service. Study payments are considered taxable income and reportable to the IRS.

If I have questions or concerns about this research study, whom can I call?

You can call us with any of your questions or concerns. If you have any illness or injury during your time on this study, you should call us promptly. Professor [redacted] is the person in charge of this research study. You can call her at telephone number [redacted]

What are my rights as a research subject?

If you choose to be in this study, you have the right to be treated with respect, including respect for your decision whether or not you wish to continue or stop being in the study. You are free to choose to stop being in the study at any time.

Choosing not to participate, or to stop participating, in this study will not result in any penalty to you or loss of benefit to which you are entitled.

Your participation in the study may be discontinued by the investigator without your consent if you are unable to adhere to the study guidelines, if you are unable to regularly attend testing or training sessions, or if your health status changes, disqualifying you from the study.

If you want to speak with someone who is not directly involved in this research, or have questions about your rights as a research subject, please contact the Institutional Review Board for the protection of human subjects [redacted]

Will my study-related information be kept confidential?

Yes, but not always. In general, we will not tell anyone any information about you. When this research is discussed or published, no one will know that you were in the study. However, laws and university rules might require us to tell certain people about you. For example, your records from this research may be seen or copied by the following people or groups:

- Representatives of the university committee and office that reviews and approves research studies, the Institutional Review Board (IRB) and Office for Protection of Research Subjects;
- Other representatives of the state and university responsible for ethical, regulatory, or financial oversight of research;
- Federal government regulatory agencies such as the Office of Human Research Protections in the Department of Health and Human Services;

If you disclose actual or suspected abuse, neglect, or exploitation of a child or a disabled or elderly adult, the researcher or members of the study staff will report the information to Child Protective Services, Adult Protective Services, and/or a law enforcement agency.

We are committed to respect your privacy and to keep your personal information confidential.

When choosing to take part in this study, you are giving us the permission to use your personal health information that includes health information in your medical records and information that can identify you. For example, personal health information may include your name, address, phone number or social security number. Your health information we may collect and use for this research includes:

- Your personal contact information,
- Results of current and past physical examinations,
- Medical history, including health information relating to your Parkinson's disease symptoms and medications.
- Health information relating to Parkinson's disease.

You are also giving permission to the following groups of people to give information about you (described above) to the researchers for this study:

- All current and previous health care providers.

The results of this study may also be used for teaching, publications, or presentations at scientific meetings.

Please note that:

- You do not have to sign this consent form. However, you will not be allowed to take part in this research study.
- You may change your mind and "take back" (revoke) this consent at any time. Even if you revoke this consent, the Principal Investigator may have already used or shared health information that was obtained about you before you revoked your consent as needed for the purpose of this study. Once you revoke this consent the Principal Investigator will not use or share again your health information. To revoke your consent for the use of your health information, you must do so in writing to: [redacted]
- Unless you revoke your consent, it will not expire.
- If there is a research-related injury, it will need to be paid for by the research participant and will not be paid for by the University of Illinois.

Optional Study Elements:

An optional aspect of the study is to allow us to take video and still images of you during the experiments for research and teaching purposes. The full image of you will be stored. There will be no personal identifiers associated to the image files apart from your image. Research purposes include the reproduction of such images in scholarly publications in print and/or electronic form. If published, your images will be viewable by anyone reading these publications, as long as the publications are available in print and/or electronic form. Please initial one of the following to indicate your choice:

_____ (initial) I agree to videotaping and storing of photographs for future research and teaching uses.

_____ (initial) I do not agree to videotaping and storing of photographs for future research and teaching uses.

Do you give us permission to contact you and provide information regarding future studies on Parkinson's?

_____ (initial) I agree to you contacting me and this is my preferred method of contact:

____

_____ (initial) I do not agree to you contacting me

Consent Summary:

I have read this consent form and the research study has been explained to me. I have been given time to ask questions, and have been told whom to contact if I have more questions. I agree to be in the research study described above. A copy of this signed consent document, information about this study and the results of any test or procedure done may be included in my medical record and may be seen by my insurance company.

A copy of this consent form will be provided to me after I sign it.

____ ________

Subject's Name (printed) and Signature Date

____ ________

Name (printed) and Signature of Person Obtaining Consent Date

University of Illinois at Urbana-Champaign
Institutional Review Board

Approved January 25, 2019
Expires January 24, 2020
IRB #16033
