# Supplementary material for: Voluntary Saccade Training Protocol in Persons With Parkinson’s Disease and Healthy Adults
Source: Front Aging Neurosci. 2019 Apr 5;11:77. doi: 10.3389/fnagi.2019.00077 (PMC6459894; doi:10.3389/fnagi.2019.00077)
Supplement: Supplementary file 2 [file Data_Sheet_2.pdf]

UNIVERSITY OF ILLINOIS
AT URBANA-CHAMPAIGN

Department of Kinesiology & Community Health

Louise Freer Hall
906 South Goodwin Avenue
Urbana, IL 61801-3895
http://www.kines.uiuc.edu
Telephone 217-300-1022

July 16th, 2018.

MEDICAL RELEASE (control group)

To the physician of____D.O.B.__________

Your patient has expressed interested in participating in a study investigating the effects of a training protocol eye saccade performance. Outcome measures include eye saccade latency prior to an involuntary/voluntary saccade, saccade duration, and saccade amplitude. The protocol involves a large TV that will display targets arranged in circles of varying sizes meant to train eye movements up to 50 degrees of the participant's field of view. Participants will be asked to make approximately 120 saccades over the course of 30 min. There will be a total of eight training sessions and two assessment sessions. As an initial measure, we screen each applicant prior to training. We ask for your assistance in approving your patient's participation in the study. Please complete the approval form if, in your medical judgment, this individual has no contraindications to participation in this study. If you have any questions please do not hesitate to contact the principal investigator in the study, [redacted]

Thank you for your help.

To be completed by the participant

Date_________________

Name____

Address____

City/State/Zip____

Phone(_____)__________________E-mail_____________________________________

Date of Birth(MM/DD/YYYY)____

Primary Diagnosis______________________________

Date of Diagnosis(MM/DD/YYYY)___________________

Emergency Contact Name____

Relationship__________________________ Phone #(_________)___________________

Do you have any allergies? (please specify) ____

Have you ever been diagnosed with the following?

Diabetes(Y/N)____(Insulin?____) Parkinson's disease____Hypertension____________

Pulmonary Disease____20/20 or Corrected Vision____ Retinal Disease(specify)______________

Glaucoma_____ Ischemic Optic Neuropathy_____ Pseudoxfoliation Syndrome_____

Ocular Surgery_____ Ocular Trauma_____ Cataracts_____ Orbital myostisis_____ Hoehn and Yahr score_____

Other (explain)____

Are you receiving outpatient therapy? (Y/N)________

Have you undergone brain surgery? (Y/N) ________

Surgeries/Dates____

____

Medications (List)

____

____

____

____

Change in use of disease modifying therapy in the last 6 months? (Y/N)________________

By signing this document, I give permission to have my physician mail a medical release form to [redacted]

Signature____Date__________________

To be completed by the participant's physician:

Participant's Name____

Diagnosis(List All)____

Mobility Impairments

____

____

Height_________ Weight_________ Pulse_________ BP_________ Sex________

Physical Exam	Normal	Abnormal	Explanation of Abnormalities
Head/Neck	______	________	_____________________________________
Eyes/Vision	______	________	_____________________________________
Ears/Hearing	______	________	_____________________________________
Heart/Lung	______	________	_____________________________________
G.U.	______	________	_____________________________________
C.N.S.	______	________	_____________________________________
Skin	______	________	_____________________________________

Dates of hospitalization over last two years with admitting diagnoses:

____

____

Significant "ABNORMAL TEST" (EKG/XRAY/LAB):_______________________________________

Medications

Has there been a change in use of disease modifying therapy in the past 6 months

YES______NO______

If yes, specify____

Initiation of medications that influence walking/mobility/coordination in the past 30 days?

YES_____NO_____

Approval for participation: YES______NO______

Comments/Restrictions:____

____

____

_____

Address:____

City:________________________________State:________________ZIP:___________________

Physician's Signature:____Date:_____________

Thank you for your time to read this summary and evaluate your patient's status for participation in this study.

Permission to release patient information to the [redacted] can be found on page 6.

MAIL TO:

[redacted]

THANK YOU FOR YOUR PARTICIPATION.
