# Supplementary material for: Voluntary Saccade Training Protocol in Persons With Parkinson’s Disease and Healthy Adults
Source: Front Aging Neurosci. 2019 Apr 5;11:77. doi: 10.3389/fnagi.2019.00077 (PMC6459894; doi:10.3389/fnagi.2019.00077)
Supplement: Supplementary file 3 [file Data_Sheet_3.pdf]

UNIVERSITYOFILLINOIS
ATURBANA-CHAMPAIGN

Department of Kinesiology & Community Health

Louise Freer Hall
906 South Goodwin Avenue
Urbana, IL 61801-3895
http://www.kines.uiuc.edu
Telephone 217-300-1022

RECEIVED
JAN 03 2019
UIUC OPRS

December 3th, 2017.

MEDICAL RELEASE

To the neurologist of ________________________________ D.O.B.__________

Your patient has expressed interest in participating in a study investigating the effects of a training protocol for voluntary eye movements in Parkinson's Disease. We are including participants with unilateral involvement only, unilateral and axial involvement, or bilateral involvement without impairment of balance. Outcome measures include saccade latency, saccade duration, and saccade amplitude in reflexive and voluntary saccades. The protocol involves a large TV screen that will display visual targets arranged in circles of varying radii to train saccades up to 50 degrees of the participant's field of view. Participants will be asked to make approximately 120 saccades over the course of 30 min. There will be a total of eight training sessions. For participants with Parkinson's disease, pre and post assessments will be taken in an "ON" and "OFF medication state". For the "OFF-state", participants will be asked to start assessments at the time of scheduled medication intake but withhold from taking the medication for two hours. During the 2-hour testing session, in the "OFF-state", participants will perform an eye tracking assessment and the Movement Disorders Society-United Parkinson's Disease Rating Scale assessment (MDS-UPDRS). Immediately after the OFF-state eye tracking and MDS-UPDRS assessments, participants will be instructed to take their next

University of Illinois at Urbana-Champaign
Institutional Review Board

Approved January 25, 2019
Expires January 24, 2020
IRB #16033

prescribed dose of medication and rest for 60 minutes before completing an "ON-state" eye tracking assessment. Please indicate whether in your medical judgment there are significant risks with a two-hour period delay in medication intake while performing assessments. If there is significant risk to your patient with disrupting medications they will be excluded from the study. Please complete the approval form in pages 4-7 if, in your medical judgment, this individual has no contraindications or serious health risks in participating in this study. Thank you for your time to read this summary and evaluate your patient's status for participation in this study. If you have any questions please do not hesitate to contact the principal investigator in the study, [redacted]

Thank you.

To be completed by the participant

Date________________

Name____

Address____

City/State/Zip____

Phone(_____)_______________E-mail_______________________________________

Date of Birth(MM/DD/YYYY)____

Primary Diagnosis_____________________________

Date of Diagnosis(MM/DD/YYYY)__________________

Emergency Contact Name____

Relationship_________________________ Phone #(_________)__________________

University of Illinois at Urbana-Champaign
Institutional Review Board

Approved January 25, 2019
Expires January 24, 2020
IRB #16033

Do you have any allergies? _________ If yes, (please specify)_________________________________

Have you ever been diagnosed with one of the following?

Diabetes(Y/N)____(Insulin?____) Parkinson's disease____Hypertension____Pulmonary Disease____

20/20 or Corrected Vision (specify)________________Retinal Disease(specify)__________________

Glaucoma_____ Ischemic Optic Neuropathy_____Pseudoexfoliation Syndrome_____ Ocular Surgery_____

Ocular Trauma________ Cataracts________ Orbital myositis_______ ________

Other neurological disorders/injuries apart from Parkinson's Disease?_________ If yes, what was the diagnosis?

____

Have you undergone brain surgery (ex: for Deep Brain Stimulation)___________________________

____

Are you receiving Outpatient Therapy YES_____NO_____

Please list any Surgeries including dates

____

____

____

____

Please list the medications that you are currently taking

____

____

____

____

____

____

University of Illinois at Urbana-Champaign
Institutional Review Board

Approved January 25, 2019
Expires January 24, 2020
IRB #16033

Has there been a change in use of medication in the last 6 months? YES_____ NO_____

Do you have a family member or caretaker that can be present and aid in transport for the "OFF medication state" assessment days? YES_____ NO_____

By signing this document, I give permission to have my neurologist mail a medical release form to Prof.

[redacted]

Signature____ Date______________

To be completed by the participant's neurologist:

Participants Name____

Diagnosis (List All)____

____

Does your patient meet the UK Brain Bank Diagnostic Criteria for PD? (y/n) ______________

Please check all the PD related impairments:

_______ unilateral involvement only

_______ unilateral and axial involvement

_______ bilateral involvement without impairment of balance

_______ Other/higher level of involvement

Height________ Weight________ Pulse________ BP________ Sex________

Physical Exam	Normal	Abnormal	Explanation of Abnormalities
Head/Neck	_____	_____	____________________
Eyes/Vision	_____	_____	____________________
Ears/Hearing	_____	_____	____________________
Heart/Lung	_____	_____	____________________
G.I.	_____	_____	____________________
C.N.S.	_____	_____	____________________
Skin	_____	_____	____________________

Dates of hospitalization over last two years with admitting diagnoses:

____

____

____

____

Significant "ABNORMAL TEST" EKG/X-RAY/LAB):______________________

Medications (please list)

____

____

____

____

____

Change in medication in the past 6 months YES______NO______ If yes, specify

____

Initiation of medications that influence walking/mobility/coordination in the past 30 days?

YES_____ NO_____

Do you expect any changes in medication in the next two months?

YES_____ NO_____

If your patient has PD, is your patient under Deep Brain Stimulation (DBS) treatment?

YES_____ NO_____

If the participant has PD, is there significant risk associated with withholding from movement related medications for two hours from the regularly scheduled intake time and resuming intake immediately after the two hour delay period?

YES_____ NO_____

Do you have any modifications to the medication intake delay time and continuation of intake after the two hour delay?

YES_____ NO_____

IF YES, please clarify:

____

____

____

____

____

Is it safe for the participant to resume medications after a two hour delay period?

YES_____ NO_____

IF NO, but there is s safe way to stop and resume medications, please provide Instructions on how stop and resume medication:

University of Illinois at Urbana-Champaign
Institutional Review Board

Approved January 25, 2019
Expires January 24, 2020
IRB #16033

Approval for participation: YES______NO______

Comments/Restrictions: ____________________

Address: ____________________

City: ____________________State: ____________ZIP:____________

Physician's Signature: ____________________

Physician's Emergency Contact Telephone Number: ____________________

Date: ____________

Permission to release patient information to the [redacted] can be found at the bottom of page 3.

PLEASE MAIL TO:

[redacted]

THANK YOU FOR YOUR PARTICIPATION.
